# A multi-site randomized trial of a clinical decision support intervention to improve problem list completeness

**DOI:** 10.1093/jamia/ocad020

**Published:** 2023-02-20

**Authors:** Adam Wright, Richard Schreiber, David W Bates, Skye Aaron, Angela Ai, Raja Arul Cholan, Akshay Desai, Miguel Divo, David A Dorr, Thu-Trang Hickman, Salman Hussain, Shari Just, Brian Koh, Stuart Lipsitz, Dustin Mcevoy, Trent Rosenbloom, Elise Russo, David Yut-Chee Ting, Asli Weitkamp, Dean F Sittig

**Affiliations:** Department of Biomedical Informatics, Vanderbilt University Medical Center, Nashville, Tennessee, USA; Department of Medicine, Vanderbilt University Medical Center, Nashville, Tennessee, USA; Department of Medicine, Brigham and Women’s Hospital, Boston, Massachusetts, USA; Digital, Mass General Brigham, Boston, Massachusetts, USA; HealthIT, Vanderbilt University Medical Center, Nashville, Tennessee, USA; Physician Informatics and Department of Internal Medicine, Penn State Health Holy Spirit Medical Center, Camp Hill, Pennsylvania, USA; Department of Medicine, Brigham and Women’s Hospital, Boston, Massachusetts, USA; Department of Medicine, Brigham and Women’s Hospital, Boston, Massachusetts, USA; Department of Medicine, Brigham and Women’s Hospital, Boston, Massachusetts, USA; Department of Medical Informatics and Clinical Epidemiology, Oregon Health and Science University, Portland, Oregon, USA; Department of Medicine, Brigham and Women’s Hospital, Boston, Massachusetts, USA; Department of Medicine, Brigham and Women’s Hospital, Boston, Massachusetts, USA; Department of Medical Informatics and Clinical Epidemiology, Oregon Health and Science University, Portland, Oregon, USA; Department of Medicine, Brigham and Women’s Hospital, Boston, Massachusetts, USA; Community Health, Mass General Brigham, Boston, Massachusetts, USA; Department of Medicine, Brigham and Women’s Hospital, Boston, Massachusetts, USA; HealthIT, Vanderbilt University Medical Center, Nashville, Tennessee, USA; Department of Biomedical Informatics, Vanderbilt University Medical Center, Nashville, Tennessee, USA; Department of Medicine, Brigham and Women’s Hospital, Boston, Massachusetts, USA; Digital, Mass General Brigham, Boston, Massachusetts, USA; Department of Biomedical Informatics, Vanderbilt University Medical Center, Nashville, Tennessee, USA; Department of Medicine, Vanderbilt University Medical Center, Nashville, Tennessee, USA; Department of Biomedical Informatics, Vanderbilt University Medical Center, Nashville, Tennessee, USA; Massachusetts General Hospital, Boston, Massachusetts, USA; Department of Biomedical Informatics, Vanderbilt University Medical Center, Nashville, Tennessee, USA; HealthIT, Vanderbilt University Medical Center, Nashville, Tennessee, USA; School of Biomedical Informatics, University of Texas Health Science Center at Houston, Houston, Texas, USA

**Keywords:** problem list, clinical decision support, electronic health record

## Abstract

**Objective:**

To improve problem list documentation and care quality.

**Materials and methods:**

We developed algorithms to infer clinical problems a patient has that are not recorded on the coded problem list using structured data in the electronic health record (EHR) for 12 clinically significant heart, lung, and blood diseases. We also developed a clinical decision support (CDS) intervention which suggests adding missing problems to the problem list. We evaluated the intervention at 4 diverse healthcare systems using 3 different EHRs in a randomized trial using 3 predetermined outcome measures: alert acceptance, problem addition, and National Committee for Quality Assurance Healthcare Effectiveness Data and Information Set (NCQA HEDIS) clinical quality measures.

**Results:**

There were 288 832 opportunities to add a problem in the intervention arm and the problem was added 63 777 times (acceptance rate 22.1%). The intervention arm had 4.6 times as many problems added as the control arm. There were no significant differences in any of the clinical quality measures.

**Discussion:**

The CDS intervention was highly effective at improving problem list completeness. However, the improvement in problem list utilization was not associated with improvement in the quality measures. The lack of effect on quality measures suggests that problem list documentation is not directly associated with improvements in quality measured by National Committee for Quality Assurance Healthcare Effectiveness Data and Information Set (NCQA HEDIS) quality measures. However, improved problem list accuracy has other benefits, including clinical care, patient comprehension of health conditions, accurate CDS and population health, and for research.

**Conclusion:**

An EHR-embedded CDS intervention was effective at improving problem list completeness but was not associated with improvement in quality measures.

## BACKGROUND AND SIGNIFICANCE

Weed^1^ first conceptualized the problem list in his landmark article “Medical Records that Guide and Teach”. He was prescient in envisioning the potential of the computerization of the problem list in the context of electronic health records (EHRs) to improve care, “Since a complete and accurate list of problems should play a central part in the understanding and management of individual patients and groups of patients, storage of this portion of the medical record in the computer should receive high priority to give immediate access to the list of problems for care of the individual patient and for statistical study on groups of patients.”

A complete patient problem list is the cornerstone of Dr Weed’s vision of the problem-oriented medical record. It serves as a valuable tool for providers assessing a patient’s clinical status and succinctly communicates this information between providers. An accurate problem list supports problem-oriented charting and can also help guide the flow of a clinical encounter, by reminding providers about important health issues to discuss or evaluate during the visit.

Complete problem lists are also important for high-quality clinical decision support (CDS). Considerable evidence exists that, when effectively designed, CDS tools can improve quality of care and patient outcomes.^2–4^ Effective CDS depends on a complete problem list, since many CDS rules require accurate, coded problem list entries.^5^ CDS systems which depend on the problem list have been developed for a wide range of purposes, including drug/problem medication alerts,^6^^,^^7^ problem-based screening reminders^8^ and management of chronic diseases.^9–21^

Problem lists are also often used for clinical research and quality measurement. Many clinical improvement and research investigations, including large genomic studies, are becoming increasingly dependent on EHR data collected during clinical care.^22–24^ For example, a genome-wide association study might correlate genomic data taken from many patients with phenotypic data derived from those patients’ EHRs.^22^^,^^25^ If EHR data are incomplete, this will lead to false negatives, which will cloud the accuracy of putative associations. To overcome this limitation, several projects, most notably the eMERGE initiative is developing EHR phenotyping algorithms that attempt to make inferences about patient’s diagnoses (or problems), even when they are missing from the problem list.^26^^,^^27^

Further, the problem list is often used for quality measurements in EHRs, including those in the CMS meaningful use/promoting interoperability incentive program.^28^ For example, a measure of retinopathy screening for patients with diabetes may depend on accurate documentation of diabetes on the problem list. If diabetes is missing from the patient’s problem list, that patient may be excluded from the measure. Conversely, if a patient has diabetes on his or her problem list, but the diabetes is in remission (or was documented erroneously), the patient may be incorrectly included in the measure, even though he or she may not be appropriate for diabetic retinopathy screening. Precision of the diagnosis is also important: even if diabetes is on the problem list, but diabetic retinopathy is not, the measure may falsely include the patient as eligible for screening, or conversely, score the patient falsely as not meeting the measure because screening appears to not have occurred. A series of recent studies have identified frequent inaccuracies in EHR-derived quality measures due to incomplete data, including problem lists.^29–38^

An accurate problem list has been associated with higher quality care in observational studies. For example, in 2005, Hartung et al^39^ found that patients with congestive heart failure (CHF) on their problem list were more likely to receive angiotensin-converting enzyme inhibitors or angiotensin-II receptor blockers than for those CHF patients without CHF listed on their problem list. A more recent thematic analysis of the literature also identified several studies which posited links between quality and problem list usage.^38^ Users of the problem list perform better on National Committee for Quality Assurance Healthcare Effectiveness Data and Information Set (NCQA HEDIS) quality measures in women’s health, depression, colon cancer screening, and cancer prevention measures, outperforming nonusers by 3.3–9.6% points on HEDIS measure group scores.^40^ Patients also increasingly view their own problem lists through patient portals,^41^ and those patients who view them report finding them helpful, so having accurate and complete problem lists may support patient comprehension.

Despite this importance, coded problem lists are often inaccurate, incomplete, cluttered, and out of date. In previous work, we showed that problem list completeness in 1 network ranged from 4.7% for renal insufficiency or failure to 50.7% for hypertension, 61.9% for diabetes,^42^ to a maximum of 78.5% for breast cancer; and other institutions have found similar results.^43–45^ Problem lists may also contain inaccurate, out-of-date or duplicative entries, causing them to become long and distracting clinicians from important problems.

Problem list completeness and use also varies dramatically by institution. For a single problem (diabetes), we found that the proportion of patients who had diabetes based on laboratory criteria had diabetes on their problem list 60.2% of the time at the lowest performing of 10 institutions, and 99.4% of the time at the highest performing institution.^46^

The causes of problem list incompleteness are myriad. In prior ethnographic work, we observed and interviewed 63 clinicians, and noted a “tragedy of the commons” occurring in many practice settings—providers reported that, frustrated with their incompleteness, they had stopped updating patient problem lists—this disuse then contributed to further decay of the problem list, causing other providers to also discontinue use.^47^

To improve problem list completeness, in prior work, we developed a series of algorithms which can identify missing problems from patient problem lists by analyzing other data in a patient’s EHR, including medications, laboratory results, and billing diagnoses. For example, if we detected a patient with a HbA1c of 9.2% who was on metformin, we inferred that they have diabetes and, if diabetes is missing from their problem list, we alerted their healthcare provider through the EHR. In a single-site randomized trial, we showed a 300% increase in problem list additions for the 17 conditions for which we had developed algorithms.^48^

Based on this prior work, we formulated 2 hypotheses: first, that the results from our single-site study could transfer to additional diseases and institutions—with CDS alerts leading to increased completeness of the problem list. Second, we further hypothesized that the CDS alerts would yield improvements in clinical quality, measured using EHR-based quality measures.

## METHODS

With funding from the National Heart, Lung and Blood Institute, we developed a 4-site randomized trial of an intervention for improving problem list completeness. Table 1 lists the 4 sites. They were selected to have a mix of vendor-developed EHRs, care settings, clinical populations, and to be geographically diverse.

**Table 1.** Sites

Site	Location	EHR	Alert setting	Alert interruptive	Alert actionable	Alert repeats	Trigger	Intervention dates
MGB^a^	Boston, MA	Epic	Outpatient	Yes	Yes	Yes, until acknowledged or resolved	Chart open	6/7/2016–6/7/2017
Holy Spirit Hospital	Camp Hill, PA	Allscripts Sunrise Acute	Inpatient	Yes	No	Yes, until resolved	Chart open	8/4/2016–5/7/2017^b^
Oregon Health and Science University	Portland, OR	Epic	Outpatient	No	Yes	Yes, until acknowledged or resolved	Navigator	6/30/2016–6/30/2017
Vanderbilt University Medical Center	Nashville, TN	Self-developed	Inpatient and outpatient	No	No	Yes, until acknowledged or resolved	Problem list activity	7/18/2016–7/18/2017

a Previously called Partners HealthCare.

b Holy Spirit Hospital transitioned from the Allscripts Sunrise EHR to Epic during the intervention period and discontinued the intervention early.

### System development

We developed and validated problem identification algorithms for 12 clinically significant heart, lung, and blood diseases:

- Asthma
- Atrial fibrillation
- Chronic obstructive pulmonary disease (COPD)
- Congestive heart failure (CHF)
- Coronary artery disease (CAD)
- Hyperlipidemia
- Hypertension
- Myocardial infarction (MI)
- Sickle cell anemia
- Sleep apnea
- Stroke
- Tuberculosis

These algorithms used a combination of laboratory results, medications, encounter diagnosis codes, and procedures to identify patients who are likely to have one of these conditions, but who do not have a relevant problem list code on their problem list. The details of the algorithms are given in Supplementary Appendix S1, and their test characteristics are described in Supplementary Table S2, based on chart reviews at Mass General Brigham (MGB) clinics prior to implementation. To maximize tolerability of the alerts, the algorithms were designed to maximize their positive predictive value (PPV) while still attaining acceptable sensitivity.

**Figure 1.**
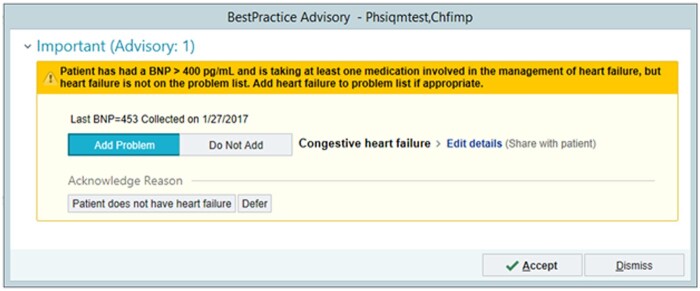
Screenshot of the IQ-MAPLE intervention, for CHF, at MGB.

We then developed a CDS alert, which fires during a clinical encounter and suggests adding the potentially missing problem to the problem list. Figure 1 shows an example alert, in Epic at MGB, for a patient who has an elevated B-type natriuretic peptide level, which suggests that the patient may have CHF^49^ and does not have CHF on his problem list. The alert suggests adding CHF, if appropriate, but also allows the user to indicate that the patient does not have CHF, which prevents the alert from firing again for this patient.

Each site used the same alerting logic and developed in-workflow CDS alerts to prompt users to add problems to the problem list. However, owing to differences in the clinical environments and EHRs at each site, they were free to tailor the presentation and workflow of the alert, following a standard framework for flexible EHR-based interventions.^50^ The flexible intervention framework is derived from the United Kingdom Medical Research Council framework, which focuses on standardizing the process and function of an intervention, rather than its form.^51^

### Study design

Each site randomized providers (physicians, physician assistants, and nurse practitioners) to the intervention or control arm, using a random number generator. Each provider had an equal probability of being assigned to either arm. Providers in the intervention arm received the alert during clinical encounters. The alert was generated and logged for providers in the control arm; however, the alert was not actually shown to the user.

Immediately after study completion, we extracted data on alert firing, alert acceptance, and problem list utilization at each site. We compared the rate of alert acceptance using a chi-squared test and the rate of problem list addition using Poisson regression.

At one site (MGB), we also evaluated the effect of the alert on clinical quality measures. This analysis was done by condition and compared patients for whom an alert was generated and displayed in the intervention arm to patients for whom an alert would have generated, but not displayed, in the control arm. This analysis was done on an intention to treat basis. For example, for the “LDL Testing” measure in the CAD condition, we calculated the proportion of patients with a low-density lipoprotein (LDL) test during the measurement period, compared across the control and intervention arms. Proportions for each quality measure were compared using a chi-squared test, with a Bonferroni correction for multiple comparisons.

The study was registered with clinicaltrials.gov prior to patient accrual, trial identifier NCT02596087, and was approved by the institutional review boards of MGB, Holy Spirit Hospital, Oregon Health and Science University, and Vanderbilt University.

## RESULTS

### Alert acceptance

Table 2 shows the proportion of missing problems added, by condition and arm. Across all sites, there were 288 832 opportunities to add a problem in the intervention arm, and the problem was added 63 777 times (overall acceptance rate of 22.1%). To isolate the effect of the intervention, we also analyzed the set of patients for whom the alert would have been presented in the control arm (our system was designed to generate and log the alert in the control arm, but not actually show it to the user). In the control arm, there were 298 817 missing problems. Of these, the problem was spontaneously added (without an alert) 6881 times (2.3%). Comparing the 2 groups, the relative ratio of alert-driven problem addition in the intervention arm was 9.6 (*P* < .0001).

**Table 2.** Proportion of missing problems added, by condition and arm

Condition	Control	Intervention	*P*
Asthma	404/23 286 = 1.7%	3164/19 309 = 16.4%	<.0001
Atrial fibrillation	173/9873 = 1.8%	1562/9774 = 16.0%	<.0001
COPD	150/10 496 = 1.4%	931/9004 = 10.3%	<.0001
CHF	88/15 197 = 0.6%	1821/15 597 = 11.7%	<.0001
CAD	236/17 319 = 1.4%	1654/15 261 = 10.8%	<.0001
Hyperlipidemia	3505/110 643 = 3.2%	36 750/112 793 = 32.6%	<.0001
Hypertension	2082/79 358 = 2.6%	14 463/79 401 = 18.2%	<.0001
Myocardial infarction	28/9650 = 0.3%	825/8912 = 9.3%	<.0001
Sickle cell	16/754 = 2.1%	136/729 = 18.7%	<.0001
Sleep apnea	93/13 228 = 0.7%	1417/10 712 = 13.2%	<.0001
Stroke	78/7962 = 1.0%	812/6347 = 12.8%	<.0001
Tuberculosis	28/1051 = 2.7%	242/993 = 24.4%	<.0001
Total	6881/298 817 = 2.3%	63 777/288 832 = 22.1%	<.0001

### Problem addition

**Figure 2.**
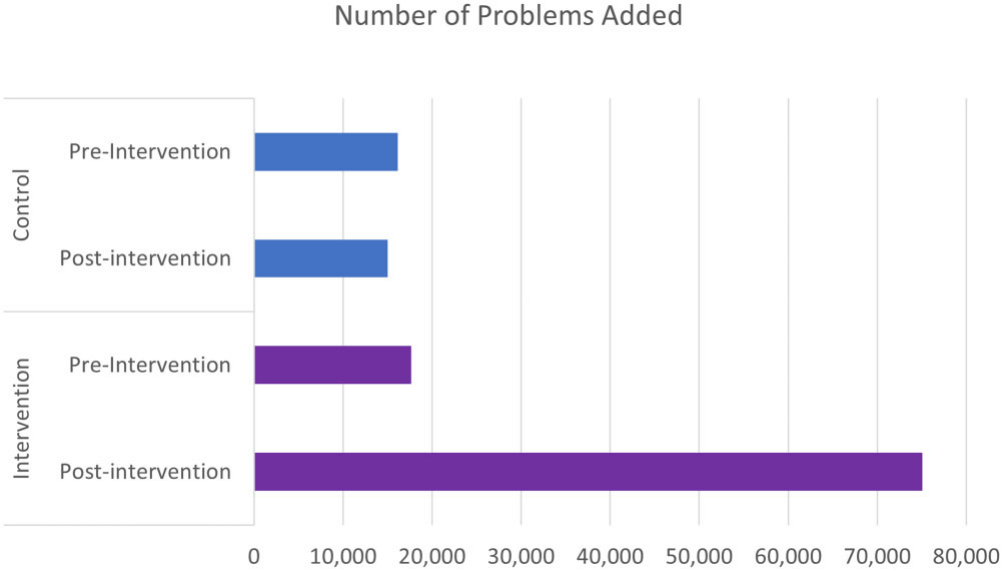
Number of problems added, by arm and period.

We calculated the total number of study problems added across all conditions and all sites, by arm (Figure 2). In the control arm, 16 132 problems were added in the preintervention period (1 year) and 15 007 were added in the postintervention period (1 year). In the intervention arm, 17 655 problems were added in the preintervention period (1 year) and 75 088 (1 year) were added in the postintervention period. Adjusting for baseline differences, the intervention arm had 4.6 times as many problems added as the control arm (*P* < .0001).

### Quality measures

Finally, we evaluated the effect of the intervention on a predetermined set of HEDIS quality measures at one site (MGB). There were no differences in quality measures between the 2 groups (Table 3). Certain HEDIS measures (such as antihyperlipidemic medicines) applied to multiple clinical conditions and were evaluated for each condition. Of the 17 condition-measure combinations, only one had a statistically significant difference; however, after Bonferroni adjustment (for multiple hypothesis testing), this difference was no longer significant (ie, at the 0.05 level, with 17 comparisons, we would expect approximately 1 false positive).

**Table 3.** Clinical outcomes

Condition	Control	Intervention	*P*
CAD			
Anti-HLD Meds	514/768 = 66.9%	545/755 = 72.2%	.030
Anti-platelet Meds	820/1105 = 74.2%	846/1130 = 74.9%	.757
BP Control	289/371 = 77.9%	287/381 = 75.3%	.456
LDL Control	369/768 = 48.0%	344/755 = 45.6%	.358
LDL Testing	459/768 = 59.8%	438/755 = 58.0%	.520
Hyperlipidemia (HLD)			
Anti-HLD Meds	24 018/28 488 = 84.3%	26 472/31 355 = 84.4%	.701
LDL Control	7412/28 488 = 26.0%	8134/31 355 = 25.9%	.839
LDL Testing	10 583/28 488 = 37.1%	11 738/31 355 = 37.4%	.474
Hypertension (HTN)			
Anti-HTN Meds	5959/7920 = 75.2%	6598/8684 = 76.0%	.276
BP control	4983/7919 = 62.9%	5388/8684 = 62.0%	.249
MI			
Anti-HLD Meds	284/457 = 62.1%	298/486 = 61.3%	.846
Anti-platelet Meds	572/755 = 75.8%	642/831 = 77.3%	.521
LDL Control	181/457 = 39.6%	179/486 = 36.8%	.418
LDL Testing	234/457 = 51.2%	237/486 = 48.8%	.494
Stroke			
Anti-HLD Meds	218/408 = 53.4%	288/480 = 60.0%	.057
Anti-platelet Meds	348/614 = 56.7%	444/726 = 61.2%	.108

## DISCUSSION

Our results demonstrate that the IQ-MAPLE intervention for problem list improvement had limited effectiveness at increasing problem list documentation, across conditions and at multiple sites. The overall acceptance rate (22.1%) is higher than those reported in many other published studies of CDS^52–54^; however, this still means that, for many patients (77.9%) for whom the alert was displayed, the provider did not act. We did not have an a priori goal for acceptance rate of the alert; however, subsequent to this trial, Vanderbilt University Medical Center (VUMC) adopted a goal of 30% acceptance for interruptive alerts. Some of the alerts were likely false positives; however, given the high PPV of our alerts identified during early testing, we expected more of them to be accepted. The causes for nonacceptance are likely multifactorial—for example, providers may not have read the alerts, or may not have thought it was their responsibility, or important, to add the problem to the problem list. Further, providers receive many other types of alerts in the EHR, and override many of them—these competing alerts may have contributed to alert fatigue, distracting providers from our problem list alerts. Based on these findings, organizations could expect that alerts such as ours would increase problem documentation significantly; however, if a higher rate of problem list completeness is required, additional strategies are necessary. For example, in a separate study, we found that an intervention where residents were paid $1.45 per chart to review patient records and confirm whether a patient had a splenectomy (from a list generated by a splenectomy-detection algorithm we developed) was at least twice as effective as a point-of-care alert.^55^

The overall impact of the alert on problem documentation was strong—leading to a 4.6-fold increase in the number of problems added in the intervention arm. This increase is statistically significant; however, it is likely that there are still many patients who have the problems of interest missing from their problem list, again suggesting a need for alternative strategies for problem list addition.

Our findings confirmed our hypothesis that the IQ-MAPLE intervention would lead to an increase in problem list additions. However, we further hypothesized that the intervention would also lead to an increase in measurable clinical quality. Unfortunately, our analysis of the HEDIS data suggests that, at least as measured by HEDIS, our intervention did not yield an increase in quality. There are several possible explanations for this. First, even after the intervention, many patients still had problem list gaps. Second, HEDIS measures may not be an accurate reflection of the true quality of care provided.^56^ Finally, the link between problem list documentation and quality may, in fact, not be very strong. MGB has CDS related to several of the HEDIS measures, and that CDS uses the problem list, as well as relevant lab results and other clinical data, to make recommendations. If problem list usage increases, this CDS may recognize more patients who have problems and offer more alerts. However, the downstream CDS at MGB had a relatively low acceptance rate, attenuating the possible causal chain from the problem list alerts to better quality through downstream CDS. Some of the measures have financial incentives, and for those population managers sometimes review gaps, and this could be an alternate mechanism for improvement, although no improvement for the metrics studied was found.

### Strengths and limitations

Our study has several strengths. It is the first multi-site, randomized study of problem list alerts done using a variety of EHRs and had a relatively effective intervention. It also had some important limitations. First, we did not assess whether the problems added from the alerts were accurate—we believe that they largely were, but there may have been some false positives. Second, although we had 4 sites, there were many differences in implementation strategy at each site, which meant we could not assess whether specific intervention characteristics (eg, inpatient vs outpatient, interruptive vs noninterruptive, and actionable vs nonactionable) made a difference. More sites or a longer time period of data collection, and a random allocation of alert features would be needed to draw these conclusions. Third, our data sharing plan did not include the sharing of additional baseline data about the rate of problem list utilization or number of encounters at each site, so we could not make direct comparisons beyond alert volume, acceptance, and the number of problems added at each site. However, since this study was a randomized controlled trial, we expect these to be well-balanced across arms at each site. Fourth, we focused, in this study, only on adding missing problems and not on removing inaccurate, unimportant, or redundant problems—problem lists frequently contain inaccurate or out-of-date problem entries, so this would be a useful topic for future research. Fifth, our study looked only at structured data already in the medical record to infer problems—by using, for example, natural language processing on notes, optical character recognition of scanned documents, and integration of external data (eg, through a health information exchange) additional problems could potentially be identified, possibly with higher specificity. Finally, more fully automated strategies, such as automatic creation and maintenance of the problem list could also be explored with a goal of reducing the overhead of maintaining the problem list for clinicians—such a strategy would need to be weighed against possible issues with accuracy, as well as the possibility that clinician curation of the problem list may have benefits as the clinician thinks through a patient’s problems.

## CONCLUSIONS

Conducting, randomized, multi-site, CDS interventions using different EHRs with different clinical workflows, upgrade cycles, patient populations, CDS governance committees, and different abilities to configure local CDS features is challenging. An EHR-embedded CDS intervention was effective at improving problem list completeness but was not associated with improvements in quality measures. The problem inference algorithms developed may have additional uses, such as improving the accuracy of clinical quality measures or EHR-based phenotyping locally or in networks such as eMERGE.

## Supplementary Material

ocad020_Supplementary_Data

## Data Availability

The data underlying this article will be shared on reasonable request to the corresponding author.
