# Supplementary material for: A multi-site randomized trial of a clinical decision support intervention to improve problem list completeness
Source: J Am Med Inform Assoc. 2023 Feb 20;30(5):899–906. doi: 10.1093/jamia/ocad020 (PMC10114117; doi:10.1093/jamia/ocad020)
Supplement: ocad020_Supplementary_Data [file ocad020_supplementary_data.zip › IQ-MAPLE Appendix A - Structured Rules.pdf]

Appendix A: IQ-MAPLE Rules for Implementation

Document Notes:

- You will need to do some localization of these rules based on the specifics of your implementation.
    - A key task will be determining what problems in your problem dictionary will "satisfy" the alert and keep it from showing. We recommend being very broad in this selection, as users become frustrated if they receive a suggestion to add a problem when something similar is already on the list.
    - Another key question is what problem (or problems) to suggest adding to the user. If your system supports it, we recommend picking one general problem to suggest as the default choice, and then offering additional more specific problems. For example, for asthma, you might suggest "asthma" as the default choice, but also offer exercise-induced asthma or cough-variant asthma as additional choices, if these are available in your problem dictionary.
    - In some cases, you may use different medications or lab results than we do at BWH. Use your best judgment to adapt the rules to your local experience.
- Another key question is where in the workflow to implement these rules. Ideally, you would implement the rules so they display at the time and place in the workflow where the user is likely to modify the problem list already, in a visible place where they are likely to be acted on, but without being overly interruptive or intrusive. We're happy to discuss options and our experience.
- For most diseases, logic is "broken up" into several different rules to simplify implementation and facilitate more relevant alert text. Many patients will meet the criteria for more than one rule for a particular disease (for example, they might meet laboratory, billing and medication criteria for that disease). When this happens, show only a single alert per disease. We recommend showing the top most alert text for each disease.
    - However, if rules for multiple <u>diseases</u> apply to a patient, show one alert for each disease. Ideally, the user could respond to all of these suggestions in a single window or area of the screen.
- Diagnosis codes "associated with a visit or hospitalization" mean that the diagnosis code was billed for an encounter or service with a CPT 99* E&M procedure code. The purpose of this restriction is to exclude diagnosis codes related to things like lab tests or imaging services, which can be less reliable in certain cases. In other cases, the rule should evaluate all billing codes.
- For a list of medications included in each class see the "Medication Classes.docx" Document
    - This may not represent a complete list of medications in each class, but represents the members of each class which have been prescribed in the BWH LMR
- Where possible, feel free to adopt existing rule criteria, groupers, classes, etc. to streamline rule building.

Please contact us with any questions or concerns about these rules – developing these alerts is a collaborative, iterative process, and we look forward to working with you and your team!

Asthma:

Asthma Rule 1:

- IF
  - Patient has >=2 billing codes in:
    - ICD-9:
      - 493.** - Asthma
    - ICD-10:
      - J45.*** - Asthma
- AND
  - Patient does not have asthma or equivalent on the problem list
- THEN
  - Alert user: Patient has at least 2 asthma diagnosis codes, but asthma is not on the problem list.
  - Offer user the option to add asthma to the problem list

Asthma Rule 2:

- IF
  - Patient has >=1 billing codes in (ICD-9: 493.**, ICD-10: J45.***) AND >=1 active medication in:
    - Inhaled Glucocorticoids (including combinations, excluding nasal sprays)
    - Leukotriene Receptor Agonists
    - Long Acting Beta2 Agonist (including combinations)
    - Mast Cell Stabilizers
    - Methylxanthine
    - Short Acting Beta2 Agonist (including combinations)
- AND
  - Patient does not have asthma or equivalent on the problem list
- THEN
  - Alert user: Patient has at least 1 asthma diagnosis code AND is taking an anti-asthmatic medication, but asthma is not on the problem list.
  - Offer user the option to add asthma to the problem list

Asthma Rule 3:

- IF
  - Patient is on Singulair (Montelukast) AND >=1 medication in:
    - Advair Diskus (Fluticasone Prop/Salmeterol)
    - Inhaled Fluticasone
    - Symbicort (Budesonide/Formoterol)
    - Albuterol
- AND
  - Patient does not have asthma or equivalent on the problem list
- THEN
  - Alert user: Patient is taking Singulair AND at least one other anti-asthmatic medication, but asthma is not on the problem list.
  - Offer user the option to add asthma to the problem list

Atrial Fibrillation:

Afib Rule 1:

- IF
  - Patient has >= 2 total billing codes at least one of which is associated with a visit or hospitalization code in :
    - ICD-9: 427.3* - Atrial fibrillation and flutter
    - ICD-10:
      - I48.0 – Paroxysmal atrial fibrillation
      - I48.1 – Persistent atrial fibrillation
      - I48.2 – Chronic atrial fibrillation
      - I48.3 – Typical atrial flutter
      - I48.4 – Atypical atrial flutter
- AND
  - Patient does not have atrial fibrillation or atrial flutter on the problem list
- THEN
  - Alert user: Patient has at least 2 atrial fibrillation diagnosis codes, but atrial fibrillation is not on the problem list.
  - Offer user the option to add atrial fibrillation or flutter to the problem list

Afib Rule 2:

- IF
  - Patient has >=1 billing code in (ICD 9: 427.3*, ICD 10: I48.**) AND is on a medication in:
    - Beta blockers (excluding combinations and eye drops)
- AND
  - Patient does not have atrial fibrillation or atrial flutter on the problem list
- THEN
  - Alert user: Patient has at least 1 atrial fibrillation diagnosis code AND is taking a β-blocker, but atrial fibrillation is not on the problem list.
  - Offer user the option to add atrial fibrillation or flutter to the problem list

Afib Rule 3:

- IF
  - Patient has at been billed for both codes:
    - ICD-9:
      - 427.31 - Atrial fibrillation
      - 427.32 – Atrial flutter
- AND
  - Patient does not have atrial fibrillation or atrial flutter on the problem list
- THEN
  - Alert user: Patient has diagnosis codes for atrial fibrillation AND atrial flutter, but neither on the problem list.
  - Offer user the option to add atrial fibrillation or flutter to the problem list

Coronary Artery Disease:

CAD Rule 1:

- IF
  - Patient has >=3 billing code associated with a hospitalization or visit in:
    - ICD-9: 414.00 – Coronary atherosclerosis of unspecified type of vessel, native or graft
    - ICD-10: I25.1* - Atherosclerotic heart disease of native coronary artery
- AND
  - Patient does not have CAD or equivalent on the problem list
- THEN
  - Alert user: Patient has at least 3 CAD diagnosis codes, but CAD is not on the problem list.
  - Offer user the option to add CAD to the problem list

CAD Rule 2:

- IF
  - Patient has had a procedure in:
    - Coronary Artery Bypass Graft
    - Placement of Stent in Coronary Artery
    - Coronary Angioplasty
- AND
  - Patient does not have CAD or equivalent on the problem list
- THEN
  - Alert user: Patient has had a CABG, PTCA, or coronary angioplasty, but CAD is not on the problem list.
  - Provide the date of procedure
  - Offer user the option to add CAD to the problem list

CAD Rule 3:

- IF
  - Patient has >=1 billing code in:
    - ICD-9:
      - V45.81 – Aortocoronary bypass status
      - V45.82 – Percutaneous transluminal coronary angioplasty status
    - ICD-10:
      - Z95.1 – Presence of aortocoronary bypass graft
      - Z98.61 – Coronary angioplasty status
      - Z95.5 – Presence of coronary angioplasty implant and graft
- AND
  - Patient does not have CAD or equivalent on the problem list
- THEN
  - Alert user: Patient has diagnosis codes for CABG or PTCA, but CAD is not on the problem list.
  - Offer user the option to add CAD to the problem list

CAD Rule 4:

- IF
  - Patient has >=1 billing code in:
    - ICD-9:
      - 414.01 – Coronary atherosclerosis of native coronary artery
      - 414.02 - Coronary atherosclerosis of autologous vein bypass graft
      - 414.04 – Coronary atherosclerosis of artery bypass graft
      - 414.8 – Other specified forms of chronic ischemic heart disease
      - 414.9 – Chronic ischemic heart disease, unspecified
    - ICD-10:
      - I25.70* – Atherosclerosis of coronary artery bypass graft(s) , unspecified, with angina pectoris
      - I25.71* - Atherosclerosis of autologous vein coronary artery bypass graft(s) with angina pectoris
      - I25.72* - Atherosclerosis of autologous artery coronary artery bypass graft(s) with angina pectoris
      - I25.73* - Atherosclerosis of nonautologous biological coronary artery bypass graft(s) with angina pectoris
      - I25.79* - Atherosclerosis of other coronary artery bypass graft(s) with angina pectoris
      - I25.8** - Other forms of chronic ischemic heart disease
      - I25.9 – Chronic ischemic heart disease, unspecified
- AND
  - Patient does not have CAD or equivalent on the problem list
- THEN
  - Alert user: Patient has at least 1 diagnosis code relating to coronary artery graft issues or ischemic heart disease, but CAD is not on the problem list.
  - Offer user the option to add CAD to the problem list

Congestive Heart Failure:

CHF Rule 1:

- IF
  - Patient has >=1 medications in:
    - Metoprolol succinate
    - Carvedilol
    - Bisoprolol
    - Atenolol
    - Spironolactone
    - Eplerenone
    - Digoxin
    - Loop diuretics
    - Direct Vasodilators
- AND
  - Patient has >=1 billing code in:
    - ICD-9: 428.** - Heart Failure
    - ICD-10: I50.** - Heart Failure
- AND
  - Patient does not have CHF or equivalent on the problem list
- THEN
  - Alert user: Patient has at least 1 heart failure diagnosis code AND is taking at least one medication involved in the management of heart failure, but heart failure is not on the problem list.
  - Offer user the option to add CHF to the problem list

CHF Rule 2:

- IF
  - Patient has >=1 billing code in:
    - ICD-9:
      - 428.2* – Systolic heart failure
      - 428.3* – Diastolic heart failure
    - ICD-10:
      - I50.2* - Systolic (congestive) heart failure
      - I50.3* – Diastolic (congestive) heart failure
      - I50.4* - Combined systolic (congestive) and diastolic (congestive) heart failure
- AND
  - Patient does not have CHF or equivalent on the problem list
- THEN
  - Alert user: Patient has at least 1 systolic heart failure or diastolic heart failure diagnosis code, but heart failure is not on the problem list.
  - Offer user the option to add CHF to the problem list

CHF Rule 3:

- IF
  - Patient has >=1 BNP lab result over 400 AND has had >=1 ntProBNP lab result >=900
- AND
  - Patient does not have CHF or equivalent on the problem list
- THEN
  - Alert user: Patient has had a BNP >400 pg/mL AND has had an ntProBNP > 900 pg/ML, but heart failure is not on the problem list.
  - Provide date and result of last BNP and ntProBNP
  - Offer user the option to add CHF to the problem list

CHF Rule 4:

- IF
  - Patient has >=1 BNP lab result over 400 and is on >=1 medication in:
    - Metoprolol succinate
    - Carvedilol
    - Bisoprolol
    - Atenolol
    - Spironolactone
    - Eplerenone
    - Digoxin
    - Loop diuretics
    - Direct Vasodilators
- AND
  - Patient does not have CHF or equivalent on the problem list
- THEN
  - Alert user: Patient has had a BNP >400 pg/mL AND is taking at least one mediation involved in the management of heart failure, but heart failure is not on the problem list.
  - Provide date and result of last BNP
  - Offer user the option to add CHF to the problem list

COPD:

COPD Rule 1:

- IF
  - Patient is >40 years of age AND DOES NOT have Smoking Status = “Never” AND has >=1 billing code in:
    - ICD-9:
      - 492.0 – Emphysematous bleb
      - 492.8 – Other emphysema
      - 496 – Chronic airway obstruction, not elsewhere classified
    - ICD-10:
      - J43.* - Emphysema
      - J44.9 – Chronic obstructive pulmonary disease, unspecified
- AND
  - Patient does not have COPD or equivalent on the problem list
- THEN
  - Alert user: Patient is >40 years old AND has at least 1 COPD diagnosis code AND may have a history of smoking, but COPD is not on their problem list.
  - Offer user the option to add COPD to the problem list

COPD Rule 2:

- IF
  - Patient is >40 years of age AND has >=3 billing codes in:
    - ICD-9: 496 – Chronic airway obstruction, not elsewhere classified
    - ICD-10: J44.9 – Chronic obstructive pulmonary disease, unspecified
- AND
  - Patient does not have COPD or equivalent on the problem list
- THEN
  - Alert user: Patient is >40 years old AND at least 3 chronic airway obstruction diagnosis codes, but COPD is not on the problem list.
  - Offer user the option to add COPD to the problem list

COPD Rule 3:

- IF
  - Patient is >40 years of age AND has >=1 billing code in (ICD-9:496, ICD-10: J44.9) AND is on at least one medication in :
    - Long Acting Beta2 Agonist (including combinations)
- AND
  - Patient does not have COPD or equivalent on the problem list
- THEN
  - Alert user: Patient is >40 years old AND is taking a long-acting beta agonist AND has at least 1 chronic airway obstruction diagnosis code, but COPD is not on the problem list.
  - Offer user the option to add COPD to the problem list

COPD Rule 4:

- IF
  - Patient is >40 years of age AND has >=1 billing code associated with a hospitalization or visit in:
    - ICD-9:
      - 491.20 – Obstructive chronic bronchitis without exacerbation
      - 491.9 – Unspecified Chronic bronchitis
      - 493.22 – Chronic obstructive asthma with (acute) exacerbation
    - ICD-10:
      - J44.0 – Chronic obstructive pulmonary disease with lower respiratory infection
      - J44.1 – Chronic obstructive pulmonary disease with (acute) exacerbation
      - J42 – Unspecified chronic bronchitis
- AND
  - Patient does not have COPD or equivalent on the problem list
- THEN
  - Alert user: Patient is >40 years old AND has at least 1 chronic bronchitis or chronic obstructive asthma diagnosis code, but COPD is not on the problem list.
  - Offer user the option to add COPD to the problem list

COPD Rule 5:

- IF
  - Patient is >40 years of age AND is on >=1 medication in:
    - Long Acting Anticholinergics
    - Short Acting Anticholinergics (excluding nasal sprays)
- AND
  - Patient is on >=1 medication in:
    - Long Acting Beta2 Agonists (including combinations)
- AND
  - Patient DOES NOT have Smoking Status = “Never”
- AND
  - Patient does not have COPD or equivalent on the problem list
- THEN
  - Alert user: Patient is >40 years old AND is taking an inhaled anticholinergic AND a long-acting beta agonist AND may have a history of smoking, but COPD is not on the problem list.
  - Offer user the option to add COPD to the problem list

COPD Rule 6:

- IF
  - Patient is >40 years of age AND is on Combivent (Ipratropium/Albuterol) AND has a history of smoking (Active or Past)
- AND
  - Patient does not have COPD or equivalent on the problem list
- THEN
  - Alert user: Patient is >40 years old AND is taking Combivent AND has a history of smoking, but COPD is not on the problem list.
  - Provide date of Combivent prescription
  - Offer user the option to add COPD to the problem list

Hypertension:

HTN Rule 1:

- IF
  - Patient has >=1 Systolic Blood Pressure >150 OR diastolic >90 AND At least one medication in:
    - ACE (including combinations)
    - ARB (including combinations)
    - Calcium Channel Blockers (including combinations)
    - Beta Blockers (including combinations, excluding eye drops)
- AND
  - Patient does not have hypertension or equivalent on the problem list
- THEN
  - Alert user: Patient has at least one blood pressure reading that is greater than 150/90 AND is taking an anti-hypertensive medication, but hypertension is not on the problem list.
  - Provide dates & value of last three blood pressure readings
  - Offer user the option to add hypertension to the problem list

HTN Rule 2:

- IF
  - Patient is on >=2 medications in:
    - ACE (including combinations)
    - ARB (including combinations)
    - Calcium Channel Blockers (including combinations)
    - Beta Blockers (including combinations, excluding eye drops)
- AND
  - Patient does not have hypertension or equivalent on the problem list
- THEN
  - Alert user: Patient is taking at least 2 anti-hypertensive medications, but hypertension is not on the problem list.
  - Provide dates & value of last three blood pressure readings
  - Offer user the option to add hypertension to the problem list

HTN Rule 3:

- IF
  - Patient has >=1 billing code associated with a visit or hospitalization in:
    - ICD-9: 401.9 – Unspecified essential hypertension
    - ICD-10: I10 – Essential (primary) hypertension
- AND
  - Patient does not have hypertension or equivalent on the problem list
- THEN
  - Alert user: Patient has at least 1 hypertension diagnosis code, but hypertension is not on the problem list.
  - Provide dates & value of last three blood pressure readings
  - Offer user the option to add hypertension to the problem list

HTN Rule 4:

- IF
  - Patient has >=1 billing code in
    - ICD-9:
      - 401.0 – Malignant essential hypertension
      - 403.90 – Hypertensive CKD, unspecified with CKD stage I – IV, or unspecified
      - 404.91 - Hypertensive heart and chronic kidney disease, unspecified, with heart failure and with chronic kidney disease stage I through stage IV, or unspecified
    - ICD-10:
      - I12.9 – Hypertensive CKD with stage I-IV CKD, or unspecified CKD
      - I13.0 – Hypertensive heart and CKD with heart failure stage I-IV CKD, or unspecified CKD
- AND
  - Patient does not have hypertension or equivalent on the problem list
- THEN
  - Alert user: Patient has at least 1 hypertension diagnosis code, but hypertension is not on the problem list.
  - Provide dates & value of last three blood pressure readings
  - Offer user the option to add hypertension to the problem list

HTN Rule 5:

- IF
  - Patient has been billed for at least two different codes in the following list (any number of times per code):
    - ICD-9:
      - 401.0 – Malignant essential hypertension
      - 401.1 – Benign essential hypertension
      - 401.9 – Unspecified essential hypertension
      - 403.9 – Unspecified hypertensive renal disease
      - 403.90 – Hypertensive CKD, unspecified with CKD stage I – IV, or unspecified
      - 403.91– Hypertensive CKD, unspecified with CKD stage V, or end stage renal disease
      - 404.91 - Hypertensive heart and chronic kidney disease, unspecified, with heart failure and with chronic kidney disease stage I through stage IV, or unspecified
    - ICD-10:
      - I10 – Essential (primary) hypertension
      - I12.0 – Hypertensive CKD, with stage 5 CKD or ESRD
      - I12.9 – Hypertensive CKD with stage I-IV CKD, or unspecified CKD
      - I13.0 – Hypertensive heart and CKD with heart failure stage I-IV CKD, or unspecified CKD
- AND
  - Patient does not have hypertension or equivalent on the problem list
- THEN
  - Alert user: Patient has at least 2 different hypertension diagnosis codes, but hypertension is not on the problem list.
  - Provide dates & value of last three blood pressure readings
  - Offer user the option to add hypertension to the problem list

Hyperlipidemia:

HLD Rule 1:

- IF
  - Patient has >=3 billing codes associated with a hospitalization or visit in:
    - ICD-9: 272.0 – pure hypercholesterolemia
    - ICD-10: E78.0 – pure hypercholesterolemia
- AND
  - Patient does not have hyperlipidemia or equivalent on the problem list
- THEN
  - Alert user: Patient has at least 3 hypercholesterolemia diagnosis codes, but hyperlipidemia is not on the problem list.
  - Provide date & value of last cholesterol reading
  - Offer user the option to add hyperlipidemia to the problem list

HLD Rule 2:

- IF
  - Patient has >=2 billing codes associated with a hospitalization or visit in:
    - ICD-9: 272.4 – other and unspecified hyperlipidemia
    - ICD-10:
      - E78.4 – Other hyperlipidemia
      - E78.5 – Hyperlipidemia, unspecified
- AND
  - Patient does not have hyperlipidemia or equivalent on the problem list
- THEN
  - Alert user: Patient has at least 2 unspecified hyperlipidemia diagnosis codes, but hyperlipidemia is not on the problem list.
  - Provide date & value of last cholesterol reading
  - Offer user the option to add hyperlipidemia to the problem list

HLD Rule 3:

- IF
  - Patient is on a Statin (including combinations)
- AND
  - Patient does not have hyperlipidemia or equivalent on the problem list
- THEN
  - Alert user: Patient is taking a statin, but hyperlipidemia is not on the problem list.
  - Provide date & value of last cholesterol reading
  - Offer user the option to add hyperlipidemia to the problem list

HLD Rule 4:

- IF
  - Patient is on a medication in:
    - Bile Acid Sequestrants
    - Fibrates
    - Ezetimibe (Zetia) (including combinations)
- AND
  - Patient has >=1 billing code in:
    - ICD-9:
      - 272.0 – pure hypercholesterolemia
      - 272.4 – other and unspecified hyperlipidemia
    - ICD-10:
      - E78.0 – pure hypercholesterolemia
      - E78.4 – Other hyperlipidemia
      - E78.5 – Hyperlipidemia, unspecified
- AND
  - Patient does not have hyperlipidemia or equivalent on the problem list
- THEN
  - Alert user: Patient has at least 1 hyperlipidemia diagnosis code AND is taking either Ezetimbe, a bile acid sequestrant, or a fibrate, but hyperlipidemia is not on the problem list.
  - Provide date & value of last cholesterol reading
  - Offer user the option to add hyperlipidemia to the problem list

Myocardial Infarction:

MI Rule 1:

- IF
  - Patient has >=2 billing codes associated with a hospitalization or visit in:
    - ICD-9:
      - 410.00 – Acute myocardial infarction of anterolateral wall, episode of care unspecified
      - 410.11 – Acute myocardial infarction of anterior wall, initial episode of care
      - 410.41 – Acute myocardial infarction of inferior wall, initial episode of care
      - 410.51 – Acute myocardial infarction of lateral wall, initial episode of care
      - 410.71 – Subendocardial wall infarction, initial episode of care
      - 410.90 – Acute myocardial infarction of unspecified site, episode of care unspecified
      - 410.92 – Acute myocardial infarction of unspecified site, subsequent episode of care
      - 412 – old myocardial infarction
    - ICD-10:
      - I21.** - ST elevation (STEMI) and non-ST elevation (NSTEMI) myocardial infarction
      - I22.** - Subsequent ST elevation (STEMI) and non-ST elevation (NSTEMI) myocardial infarction
      - I25.2 – Old myocardial infarction
- AND
  - Patient does not have myocardial infarction or equivalent on the problem list
- THEN
  - Alert user: Patient has at least 2 myocardial infarction diagnosis codes, but myocardial infarction is not on the problem list.
  - Offer user the option to add myocardial infarction to the problem list

MI Rule 2:

- IF
  - Patient has >=3 billing codes in:
    - ICD-9:
      - 410.60 – True posterior wall infarction, episode of care unspecified
      - 410.70 – Subendocardial infarction, episode of care unspecified
      - 410.90 – Acute myocardial infarction of unspecified site, episode of care unspecified
      - 410.92 – Acute myocardial infarction of unspecified site, subsequent episode of care
      - 412 – old myocardial infarction
    - ICD-10:
      - I21.** - ST elevation (STEMI) and non-ST elevation (NSTEMI) myocardial infarction
      - I22.** - Subsequent ST elevation (STEMI) and non-ST elevation (NSTEMI) myocardial infarction
      - I25.2 – Old myocardial infarction
- AND
  - Patient has >=1 CK lab result >400 AND has >=1 Abnormal TropT/I result
- AND
  - Patient does not have myocardial infarction or equivalent on the problem list
- THEN
  - Alert user: Patient has at least 3 myocardial infarction diagnosis codes AND has at least 1 serum CK >400 U/L AND has at least one abnormal troponin, but myocardial infarction is not on the problem list.
  - Provide date and value of last CK and troponin results
  - Offer user the option to add myocardial infarction to the problem list

MI Rule 3:

- IF
  - Patient is on Aspirin (excluding combinations) AND a Beta Blocker (including combinations) OR Nitrate (excluding combinations) AND has a billing code in:
    - ICD-9:
      - 412 – old myocardial infarction
      - 410.60 – True posterior wall infarction, episode of care unspecified
      - 410.70 – Subendocardial infarction, episode of care unspecified
      - 410.90 – Acute myocardial infarction of unspecified site, episode of care unspecified
      - 410.92 – Acute myocardial infarction of unspecified site, subsequent episode of care
    - ICD-10:
      - I21.** - ST elevation (STEMI) and non-ST elevation (NSTEMI) myocardial infarction
      - I22.** - Subsequent ST elevation (STEMI) and non-ST elevation (NSTEMI) myocardial infarction
      - I25.2 – Old myocardial infarction
- AND
  - Patient does not have myocardial infarction or equivalent on the problem list
- THEN
  - Alert user: Patient has at least 1 myocardial infarction diagnosis code AND is taking aspirin AND either a β-blocker or nitrate, but myocardial infarction is not on the problem list.
  - Offer user the option to add myocardial infarction to the problem list

MI Rule 4:

- IF
  - Patient is on Aspirin (excluding combinations) AND a Beta Blocker (including combinations) OR Nitrate (excluding combinations) AND has >=1 Abnormal tropI/T
- AND
  - Patient does not have myocardial infarction or equivalent on the problem list
- THEN
  - Alert user: Patient is taking aspirin AND either a β-blocker or nitrate AND has at least 1 abnormal troponin, but myocardial infarction is not on the problem list.
  - Provide date and value of last troponin result
  - Offer user the option to add myocardial infarction to the problem list

Sickle Cell:

SC Rule 1:

- IF
  - Patient has an HbSS result > 0
- AND
  - Patient does not have sickle cell disease or trait on the problem list
- THEN
  - Alert user: Patient has a hemoglobin SS laboratory result >0, but sickle cell disease or trait is not on the problem list.
  - Provide date and result of last Hb-SS
  - Offer user the option to add sickle cell disease or trait to the problem list

SC Rule 2:

- IF
  - Patient has >=5 billing codes associated with a hospitalization or visit in:
    - ICD-9: 282.60 – Sickle-cell disease, unspecified
    - ICD-10: D57.1 – Sickle cell disease without crisis
- AND
  - Patient does not have sickle cell disease or trait on the problem list
- THEN
  - Alert user: Patient has at least 5 sickle cell disease diagnosis codes, but sickle cell disease or trait is not on the problem list.
  - Offer user the option to add sickle cell disease or trait to the problem list

SC Rule 3:

- IF
  - Patient has >=1 billing code associated with a hospitalization or visit in:
    - ICD-9:
      - 282.61 – Hb-SS disease without crisis
      - 282.63 – Sickle cell/Hb-C disease without crisis
      - 282.64 – Sickle-cell/Hb-C disease with crisis
      - 282.69 – Other sickle-cell disease with crisis
    - ICD-10:
      - D57.0* - Hb-SS disease with crisis
      - D57.2* - Sickle-cell/Hb-C disease with crisis
      - D57.8** - Other sickle-cell disorders
- AND
  - Patient does not have sickle cell disease or trait on the problem list
- THEN
  - Alert user: Patient has at least 1 sickle cell disease diagnosis code with or without crisis, but sickle cell disease or trait is not on the problem list.
  - Offer user the option to add sickle cell disease or trait to the problem list

SC Rule 4:

- IF
  - Patient has been billed for at least two different codes in the following list (any number of times per code):
    - ICD-9:
      - 282.5 – Sickle cell trait
      - 282.6* - Sickle cell disease
    - ICD-10:
      - D57.0* - Hb-SS disease with crisis
      - D57.1 – Sickle cell disease without crisis
      - D57.2* - Sickle-cell/Hb-C disease with crisis
      - D57.3 – Sickle cell triat
      - D57.8** - Other sickle-cell disorders
- AND
  - Patient does not have sickle cell disease or trait on the problem list
- THEN
  - Alert user: Patient has at least 2 different sickle cell disease or trait diagnoses codes, but sickle cell disease or trait is not on the problem list.
  - Offer user the option to add sickle cell disease or trait to the problem list

SC Rule 5:

- IF
  - Patient has >=3 billing codes associated with a hospitalization or visit in:
    - ICD-9: 282.5 – Sickle cell trait
    - ICD-10: D57.3 – Sickle cell trait
- AND
  - Patient does not have sickle cell disease or trait on the problem list
- THEN
  - Alert user: Patient has at least 3 sickle cell trait diagnosis codes, but sickle cell disease or trait is not on the problem list.
  - Offer user the option to add sickle cell disease or trait to the problem list

Sleep Apnea:

OSA Rule 1:

- IF
  - Patient has >=2 billing codes in:
    - ICD-9:
      - 327.20 – Organic sleep apnea, unspecified
      - 327.21 – Primary central sleep apnea
      - 327.23 – Obstructive sleep apnea (adult) (pediatric)
      - 327.26 – Sleep related hypoventilation/hypoxemia in conditions classifiable elsewhere
      - 327.27 – Central sleep apnea in conditions classified elsewhere
      - 780.51: insomnia with sleep apnea, unspecified
      - 780.53: hypersomnia with sleep apnea, unspecified
      - 780.57: unspecified sleep apnea
    - ICD-10: G47.3* - Sleep apnea
- AND
  - Patient does not have sleep apnea or equivalent on the problem list
- THEN
  - Alert user: Patient has at least 2 sleep apnea diagnosis codes, but sleep apnea is not on the problem list.
  - Offer user the option to add sleep apnea to the problem list

Stroke:

Stroke Rule 1:

- IF
  - Patient has >=1 billing code in:
    - ICD-9:
      - V12.54 – Personal history of transient ischemic attack, and cerebral infarction without residual effects
      - 997.02 – Iatrogenic cerebrovascular infarction or hemorrhage
      - 434.01 – Cerebral thrombosis with cerebral infarction
    - ICD-10:
      - Z86.73 – Personal history of transient ischemic attack (TIA), and cerebral infarction without residual effects
      - I97.810 – Intraoperative cerebrovascular infarction during cardiac surgery
      - I97.811 – Intraoperative cerebrovascular infarction during other surgery
      - I97.820 – Postprocedural cerebrovascular infarction during cardiac surgery
      - I97.821 – Postprocedural cerebrovascular infarction during other surgery
      - I63.3** - Cerebral infarction due to thrombosis of cerebral arteries
- AND
  - Patient does not have stroke or equivalent on the problem list
- THEN
  - Alert user: Patient has at least 1 stroke diagnosis code, but stroke is not on the problem list.
  - Offer user the option to add stroke to the problem list

Stroke Rule 2:

- IF
  - Patient has >=1 billing code associated with a hospitalization or visit in:
    - ICD-9:
      - 434.11 – Cerebral embolism with cerebral infarction
      - 434.91 – Cerebral artery occlusion, unspecified with cerebral infarction
    - ICD-10:
      - I63.4** - Cerebral infarction due to embolism of cerebral arteries
      - I63.5** - Cerebral infarction due to unspecified occlusion or stenosis of cerebral arteries
- AND
  - Patient does not have stroke or equivalent on the problem list
- THEN
  - Alert user: Patient has at least 1 stroke diagnosis code related to occlusion of the cerebral arteries, but stroke is not on the problem list.
  - Offer user the option to add stroke to the problem list

Stroke Rule 3:

- IF
  - Patient has >=2 billing codes in:
    - ICD-9:
      - 433.01 – Occlusion and stenosis of basilar artery with cerebral infarction
      - 433.11 – Occlusion and stenosis of carotid artery with cerebral infarction
      - 433.21 – Occlusion and stenosis of vertebral artery with cerebral infarction
      - 433.31 – Occlusion and stenosis of multiple and bilateral precerebral arteries with cerebral infarction
    - ICD-10:
      - I63.22 - Cerebral infarction due to unspecified occlusion or stenosis of basilar arteries
      - I63.23* - Cerebral infarction due to unspecified occlusion or stenosis of carotid arteries
      - I63.21* - Cerebral infarction due to unspecified occlusion or stenosis of vertebral arteries
      - I63.59 – Cerebral infarction due to unspecified occlusion or stenosis of other cerebral artery
- AND
  - Patient does not have stroke or equivalent on the problem list
- THEN
  - Alert user: Patient has at least 2 stroke diagnosis codes relating to occlusion and stenosis of an artery, but stroke is not on the problem list.
  - Offer user the option to add stroke to the problem list

Stroke Rule 4:

- IF
  - Patient has >=3 billing codes in:
    - ICD-9:
      - 434.11 – Cerebral embolism with cerebral infarction
      - 434.91 – Cerebral artery occlusion, unspecified with cerebral infarction
    - ICD-10:
      - I63.4** - Cerebral infarction due to embolism of cerebral arteries
      - I63.5** - Cerebral infarction due to unspecified occlusion or stenosis of cerebral arteries
- AND
  - Patient does not have stroke or equivalent on the problem list
- THEN
  - Alert user: Patient has at least 3 stroke diagnosis codes related to occlusion of the cerebral arteries, but stroke is not on the problem list.
  - Offer user the option to add stroke to the problem list

Tuberculosis:

TB Rule 1:

- IF
  - Patient has >=1 positive IGR Assay test result
- AND
  - Patient does not have tuberculosis or equivalent on the problem list
- THEN
  - Alert user: Patient has at least one positive interferon gamma assay test result, but tuberculosis is not on the problem list.
  - Provide date of positive INF-gamma result
  - Offer user the option to add tuberculosis to the problem list

TB Rule 2:

- IF
  - Patient has >= 2 billing codes associated with a hospitalization or visit in:
    - ICD-9: 011.90 – Pulmonary tuberculosis, unspecified, unspecified
    - ICD-10: A15.0 – Tuberculosis of long
- AND
  - Patient does not have tuberculosis or equivalent on the problem list
- THEN
  - Alert user: Patient has at least 2 pulmonary tuberculosis diagnosis codes, but tuberculosis is not on the problem list.
  - Offer user the option to add tuberculosis to the problem list

TB Rule 3:

- IF
  - Patient has >=1 billing code in:
    - ICD-9:
      - V12.01 – Personal history of tuberculosis
    - ICD-10:
      - Z86.11 – Personal history of tuberculosis
- AND
  - Patient does not have tuberculosis or equivalent on the problem list
- THEN
  - Alert user: Patient has at least 1 personal history of tuberculosis diagnosis code, but tuberculosis is not on the problem list.
  - Offer user the option to add tuberculosis to the problem list

TB Rule 4:

- IF
  - Patient has >= 2 billing codes in:
    - ICD-9:
      - 010.00 – Tuberculous pleurisy in primary progressive tuberculosis, unspecified
      - 010.90 – Primary tuberculosis infection, unspecified, unspecified
      - 011.10 – Tuberculosis of lung, nodular, unspecified
    - ICD-10: A15.7 – Primary respiratory tuberculosis
  -
- AND
  - Patient does not have tuberculosis or equivalent on the problem list
- THEN
  - Alert user: Patient has at least 2 unspecified tuberculosis diagnosis codes, but tuberculosis is not on the problem list.
  - Offer user the option to add tuberculosis to the problem list

TB Rule 5:

- IF
  - Patient has >=1 billing code associated with a hospitalization or visit in:
    - ICD-9:
      - 010.00 – pleurisy in primary progressive tuberculosis, unspecified
      - 011.00 – Tuberculosis of lung, infiltrative, unspecified
    - ICD-10: A15.7 – Primary respiratory tuberculosis
- AND
  - Patient does not have tuberculosis or equivalent on the problem list
- THEN
  - Alert user: Patient has at least 1 unspecified tuberculosis diagnosis code, but tuberculosis is not on the problem list.
  - Offer user the option to add tuberculosis to the problem list

TB Rule 6:

- IF
  - Patient is on >=1 medication in:
    - Isonaizid
    - Pyrazinamide
- AND
  - Patient does not have tuberculosis or equivalent on the problem list
- THEN
  - Alert user: Patient is taking an anti-mycobacterial medication, but tuberculosis is not on the problem list.
  - Offer user the option to add tuberculosis to the problem list
