# Supplementary material for: A multi-site randomized trial of a clinical decision support intervention to improve problem list completeness
Source: J Am Med Inform Assoc. 2023 Feb 20;30(5):899–906. doi: 10.1093/jamia/ocad020 (PMC10114117; doi:10.1093/jamia/ocad020)
Supplement: ocad020_Supplementary_Data [file ocad020_supplementary_data.zip › IQ-MAPLE Appendix Table A1 - Test Characteristics of the IQ-MAPLE Algorithms.pdf]

Appendix Table A1: Test Characteristics of IQ-MAPLE algorithms

**Condition**	**Sensitivity**	**Specificity**	**PPV**	**NPV**
Asthma	78.4%	99.4%	95.1%	96.9%
Atrial fibrillation	67.3%	99.7%	94.7%	97.6%
COPD	68.8%	99.5%	88.0%	98.4%
CHF	58.4%	99.6%	90.7%	97.0%
CAD	76.4%	99.4%	93.2%	97.5%
Hyperlipidemia	84.7%	98.1%	96.2%	91.8%
Hypertension	71.0%	97.3%	93.0%	86.7%
MI	48.3%	99.3%	81.4%	96.9%
Sickle Cell	89.3%	100.0%	99.1%	100.0%
Sleep Apnea	87.1%	99.9%	97.2%	99.5%
Stroke	65.1%	99.7%	90.6%	98.6%
Tuberculosis	74.8%	100.0%	88.7%	99.9%
